# ZW Sex Chromosome Differentiation in Palaeognathous Birds Is Associated with Mitochondrial Effective Population Size but Not Mitochondrial Genome Size or Mutation Rate

**DOI:** 10.1093/gbe/evaf005

**Published:** 2025-01-23

**Authors:** Brooke Weinstein, Zongji Wang, Qi Zhou, Scott William Roy

**Affiliations:** Department of Molecular and Cell Biology, University of California-Merced, Merced, CA 95343, USA; Institute of Animal Sex and Development, Zhejiang Wanli University, Ningbo, Zhejiang 315100, China; Institute of Animal Sex and Development, Zhejiang Wanli University, Ningbo, Zhejiang 315100, China; Evolutionary & Organismal Biology Research Center, School of Medicine, Zhejiang University, Hangzhou, Zhejiang 310058, China; Department of Molecular and Cell Biology, University of California-Merced, Merced, CA 95343, USA; Department of Biology, San Francisco State University, San Francisco, CA 94117, USA

**Keywords:** evolution of complexity, purifying selection, Hill–Robertson interference

## Abstract

Eukaryotic genome size varies considerably, even among closely related species. The causes of this variation are unclear, but weak selection against supposedly costly “extra” genomic sequences has been central to the debate for over 50 years. The mutational hazard hypothesis, which focuses on the increased mutation rate to null alleles in superfluous sequences, is particularly influential, though challenging to test. This study examines the sex chromosomes and mitochondrial genomes of 15 flightless or semiflighted palaeognathous bird species. In this clade, the nonrecombining portion of the W chromosome has independently expanded stepwise in multiple lineages. Given the shared maternal inheritance of the W chromosome and mitochondria, theory predicts that mitochondrial effective population size (*N*_e_) should decrease due to increased Hill–Robertson interference in lineages with expanded nonrecombining W regions. Our findings support the extent of the nonrecombining W region with three indicators of reduced selective efficiency: (i) the ratio of nonsynonymous to synonymous nucleotide changes in the mitochondrion, (ii) the probability of radical amino acid changes, and (iii) the number of ancient, W-linked genes lost through evolution. Next, we tested whether reduced *N*_e_ affects mitochondrial genome size, as predicted by weak selection against genome expansion. We find no support for a relationship between mitochondrial genome size and expanded nonrecombining W regions, nor with increased mitochondrial mutation rates (predicted to modulate selective costs). These results highlight the utility of nonrecombining regions and mitochondrial genomes for studying genome evolution and challenge the general idea of a negative relation between the efficacy of selection and genome size.

**Significance**

Explaining the striking variation in eukaryotic genome size and complexity has been a long-standing challenge, primarily due to the need for well-controlled experiments. Using the shared maternal inheritance of W chromosomes and mitochondrial genomes, we explore a novel avenue for studying genome evolution. Our investigation of 15 palaeognathous bird species, which have experienced stepwise recombination suppression between the Z and W chromosomes, introduces a powerful framework for unraveling genome structure evolution and confirms a compelling theoretical prediction: increased sex chromosome differentiation correlates with decreased mitochondrial selective strength and efficiency. However, we find no evidence of mitochondrial genome expansion under these conditions or with changes in mutation rate, calling into question that genome size and complexity are driven by differential selective efficiency on nearly neutral superfluous genomic sequences.

## Introduction

Nuclear and organellar genomes exhibit remarkable diversity in content and structure across eukaryotes, characterized by significant variation in gene numbers, introns, gene copies, and intergenic DNA (Smith and Keeling 2015). For over five decades, the hypothesis that seemingly superfluous genomic elements persist in populations due to lack of weak or absent selection (Ohta 1973, 1992; Doolittle 1978) has been central in debates about the origins of genome size (GS) and complexity (Lynch 2007). Despite this long-standing discussion, the issue remains unresolved, mainly due to technical complications, including challenges in estimating key parameters such as the strength of selection. An alternative approach compares species with varying effective population sizes (*N*_e_). *N*_e_ represents the theoretical number of individuals in an ideal population experiencing genetic drift at a rate equivalent to the actual population, accounting for ecological, demographic, and genomic complexities (Charlesworth 2009). Given that the relative influence of natural selection versus genetic drift varies with *N*_e_, if genome expansions are generally slightly harmful, they should be more likely to persist when *N*_e_ is small (Lynch and Conery 2003; Lynch 2007).

The most extensively developed version of the conjecture that GS and complexity differences reflect differences in the efficacy of selection is the mutational hazard hypothesis (MHH; Lynch and Conery 2003). The MHH posits that alleles containing various types of additional sequences (introns, extra gene copies, repetitive elements, etc.) will tend to have a higher rate of mutation to null alleles, manifesting as a small nonzero cost relative to alleles lacking the addition sequence. Thus, populations with small *N*_e_ will disproportionately accumulate genomic insertions. In their original analysis, Lynch and Conery examined 43 genomes spanning prokaryotes, protists, fungi, plants, and animals. They estimated nucleotide silent site diversity, π, predicted to equal 4*N*_e_*u* or 2*N*_e_*u* (for diploids and haploids, respectively, where *u* represents the assumed constant per nucleotide mutation rate), and found that *N*_e_*u* explained a significant portion of the observed variation in nuclear GS. However, objections swiftly emerged on theoretical and methodological grounds.

Charlesworth and Barton (2004) highlighted challenges in accurately measuring *N*_e_ and confounding effects with other aspects of organismal biology (such as development rate and body size). Additionally, questions arose about whether microbes with large global *N*_e_ might experience more substantial fitness effects from genome expansions than multicellular organisms with smaller *N*_e_ (Batut et al. 2014). Furthermore, correcting for shared phylogenetic history revealed that the perceived association between *N*_e_ and GS vanished (Whitney and Garland 2010). Recent tests of the MHH have yielded elusive and contradictory results, primarily due to correlated evolutionary changes and ongoing debates over appropriate measures and definitions of *N*_e_ (Lefébure et al. 2017; Roddy et al. 2021).

Concerns about the challenges in measuring *N*_e_ directly in natural populations have been raised, so most studies rely on genetic or life history characteristics as proxies (Waples et al. 2013; James et al. 2017). Beyond demographic factors, *N*_e_ at a genomic locus is influenced by the number of sites genetically linked to it. Deleterious mutations at these linked sites tend to remove chromosomes from the population, effectively decreasing *N*_e_ (Charlesworth 2009). This is especially evident in differentiated nonrecombining sex chromosomes (e.g. W and Y chromosomes), where newly sex-linked nonrecombining regions rapidly accumulate deleterious mutations and lose genes (Charlesworth and Charlesworth 2000). Consequently, comparing genome evolution across related lineages with varying degrees of linkage is a promising approach for testing the MHH.

A compelling model system for investigating whether species with reduced *N*_e_ and lower efficacy of selection evolve larger genomes comes from palaeognathous bird sex chromosomes. While most mammals share the same XY chromosome pair, nearly all birds possess a sex-linked region within the ZW sex chromosomes. In most birds and all mammals, most of the sex chromosome pair is differentiated. However, Palaeognathae, the earliest diverging branch of birds, represent an exception. Palaeognathae, including flightless ratites and semiflighted tinamous, diverged from other birds over 110 million years ago (Jarvis et al. 2014). Intriguingly, the Z and W chromosomes remain homomorphic in many palaeognathous species, associated with continued recombination in ZW females. Yet in multiple lineages, the nonrecombining sex-linked portion of the chromosome has independently expanded due to stepwise, parallel recombination suppression (Wang et al. 2022).

The power of this model system for studying GS evolution stems from the peculiar inheritance of W chromosomes. Analogous to Y chromosomes in mammals, differentiated regions of W chromosomes in birds are hemizygous and thus do not undergo recombination (Charlesworth and Charlesworth 2000). Since the sex-specific (nonrecombining) portion of the W chromosome strictly follows maternal inheritance, it is expected to experience complete genetic linkage with the entire mitochondrial genome (also maternally inherited). Rare or no recombination occurs within either genomic element. Thus, reassortment between them is expected to be absent or minimal, resulting in increased linkage predicted to amplify the effects of background selection and hitchhiking, leading to increased Hill–Robertson interference (HRI) and reduced *N*_e_ of the mitochondrial genome (Hill and Robertson 1966; Felsenstein 1974; Santiago and Caballero 1995; McVean and Charlesworth 2000; Comeron et al. 2008; Charlesworth and Jensen 2021). Some empirical support for reduced *N*_e_ during the expansion of the sex-specific W region comes from observations of decreased nucleotide diversity in mitochondrial genes from ZW species (Berlin et al. 2007). Parallel evolutionary ZW differentiation across diverse palaeognathous lineages, for which genomic data are available, presents a rare opportunity to test the hypothesis that diminished selective efficiency leads to larger genomes.

If the MHH holds, it should apply to all genomic features, including organelle GS. However, Lynch et al. (2006) observed animals and land plants with similar *N*_e_ but dramatically different mitochondrial GS and proposed an alternative hypothesis: organelle genome variation results from differences in *u* rather than *N*_e_, as previously suggested for nuclear genomes. According to their argument, for mitochondria, the strength of selection, rather than its efficacy, primarily drives GS and complexity evolution, with a lower *u* creating a more permissive environment for accumulating additional hazardous DNA.

Similarly, as with Lynch and Conery (2003) deployment of the MHH, support from subsequent investigations into Lynch et al. (2006) proposal for organelle genomes have yielded mixed support. Despite decades of research, the evolutionary forces governing GS variation remain unclear. For a comprehensive review and discussion of competing hypotheses, refer to Blommaert (2020) and Galtier (2024) for nuclear genomes and Smith (2016) for organelle genome evolution. Before testing the two alternative predictions—GS expansion under either decreased *N*_e_ or decreased *u*—we first confirmed the reduced *N*_e_ of the mitochondrial genome under expanded ZW differentiation. We then examined whether increases in mitochondrial GS variation are associated with enhanced drift in species with expanded regions of ZW recombination suppression or decreased *u*.

## Results

**Fig. 1.**
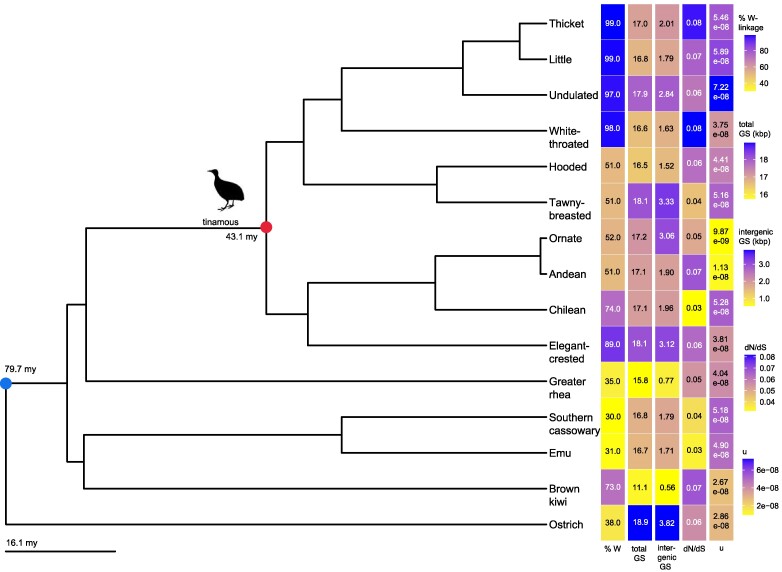
Palaeognathae phylogeny and raw mitochondrial trait data. Chronogram of the 15 palaeognathous bird species analyzed. Heat maps depict the raw trait data. GS represents the total mitochondrial GS in kilobase pairs, while intergenic GS is the total GS minus the coding region, tRNA, and rRNA genes. *u* is the estimated mutation rate per generation per base pair. The red dot denotes the tinamou order/family. Branch lengths are in millions of years.

Differentiated W-linked genomic regions, inherited exclusively from mother to daughter without recombination, mirror the inheritance pattern of mitochondrial genomes and are completely genetically linked. The expansion of the nonrecombining W-linked region is thus expected to correlate with heightened HRI effects across both regions and the mitochondrial genome, provided the expanded nonrecombining segments harbor an increased complement of functional sequences. To test this hypothesis, we obtained mitochondrial genomes and the percentage of the sex chromosome pair that exhibits differentiation, along with a maximum likelihood chronogram estimated from whole-genome noncoding sequences, for 15 species of palaeognathous birds, all previously generated by Wang et al. (2022). Among these species, differentiated ZW regions vary widely in size, from 30% to 99% of the entire chromosome, with a relative variation (the percentage ratio of the range to the average value) of 106%. Our initial focus was on mitochondrial genes, so we created a concatenated alignment of the 13 protein-coding sequences across the 15 genomes. We found that the relative percent range in the protein-coding sequence (CDS) size is just 9.3% between species (ranging from 10.0 to 11.0 kb). However, the size of intergenic regions varies considerably more, with a relative variation of 153% (from 564 to 3,815 base pairs). Overall, total mitochondrial GS has a relative range of 18.6% (from 15.8 to 18.9 kb; Fig. 1).

### Diverse Evidence Suggests That ZW Sex Chromosome Differentiation Impacts the Effective Population Size of W Chromosomes and Mitochondrial Genomes

**Fig. 2.**
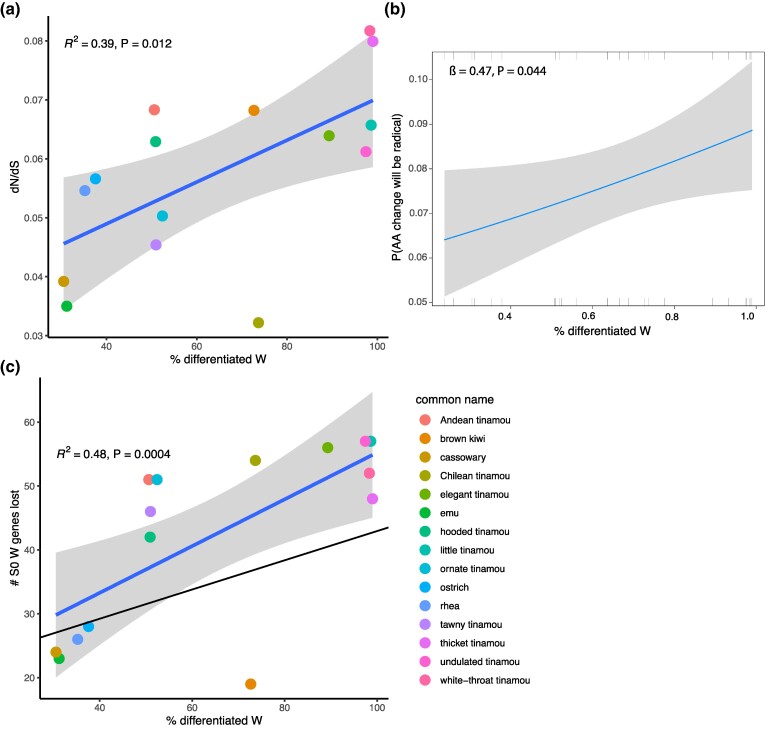
Confirmation of reduced selective efficiency due to increased recombination suppression. a) A significant positive association exists between d_N_/d_S_ and % ZW linkage. b) The predicted probability of an amino acid change being radical (estimated using binomial logistic regression) increases with increasing % ZW linkage. Tick marks represent observations of conservative (0; shown on the bottom axis) and radical (1; indicated on the top axis) amino acid changes. *β* represents the logit coefficient, which is the change in the log odds of an amino acid change occurring being radical associated with every 1% increase in ZW linkage. c) A robust positive association is observed between the number of ancient W-linked S0 genes lost and % ZW linkage. The blue line shows the regression coefficient (*β*) from OLS regression, and the black line is from PGLS regression in 15 species of palaeognathous birds.

We first sought to test whether the predicted relationship between the extent of ZW differentiation and *N*_e_ holds. To assess the effect of ZW differentiation on mitochondrial gene evolution, we reconstructed branch-specific ratios of nonsynonymous changes (d_N_; subject to selection) to synonymous changes (d_S_; presumed neutral) across the phylogenetic tree. Previous theoretical and empirical work shows that, under certain assumptions, the greater influence of genetic drift under reduced *N*_e_ can lead to an overall increased fixation of deleterious nonsynonymous variants. Consequently, d_N_/d_S_ is expected to negatively correlate with *N*_e_ (Ohta 1992; Woolfit and Bromham 2005; Weyna and Romiguier 2021), resulting in a positive relationship between the extent of recombination suppression on the W chromosome and d_N_/d_S_. Consistent with this prediction, our ordinary least square (OLS) regression analysis robustly confirms a positive association between the degree of ZW differentiation and d_N_/d_S_ in mitochondrial genes (*β* = 0.035, *P* = 0.012; Fig. 2a). To ensure that the observed variation in d_N_/d_S_ is not solely due to differences in synonymous branch length, we tested for a correlation between d_N_/d_S_ and d_S_. We found no evidence of such an association (*β* = 0.0009, *P* = 0.962). Thus, as predicted, the size of the ZW differentiated region significantly influences the mutation-normalized probability of fixation of nonsynonymous changes.

It is important to note that we did not apply phylogenetic correction in the model testing d_N_/d_S_ as affected by % ZW differentiation. The necessity and value of phylogenetic correction arise from character states that apply to a given extant or ancestral taxon. These character states exhibit phylogenetic inertia over time, remaining unchanged unless altered by evolutionary processes (Felsenstein 1985). However, d_N_/d_S_ is not a character state but a measure of evolutionary change estimated from comparing a taxon and its direct ancestor. Consequently, the concept of phylogenetic inertia does not apply to d_N_/d_S_ (or the occurrence of radical amino acid changes discussed below) in the same way.

Improper application of phylogenetic regression can lead to suboptimal statistical performance, especially when all the phylogenetic inertia is present in the predictor variable(s), as noted by Rohlf (2006). Phylogenetic signal is generally assessed using Pagel's *λ*, which ranges from zero to one, where values close to zero suggest the trait evolves independently of phylogeny, and values near one indicate the trait evolves following Brownian motion along the branches. Within our data, all continuous variables exhibit moderate to strong phylogenetic signals, represented by Pagel's *λ*, except for d_N_/d_S_ and the amount of intergenic DNA (left side of Table 1). Still, to be cautious, we followed Revell’s (2010) recommendation to assess the appropriateness of phylogenetic regression by testing whether model residuals contain a phylogenetic signal, representing unexplained variation in the model associated with shared evolutionary history. Again, while most regressions showed substantial signal, d_N_/d_S_, distributed by % ZW, did not (right side of Table 1). Revell (2010) also demonstrated that phylogenetic correction is inappropriate and can be misleading under such circumstances. Therefore, we corrected for phylogenetic inertia using phylogenetic generalized least squares (PGLS) for all linear regressions except for the contribution of ZW differentiation to the d_N_/d_S_ ratio.

**Table 1:** Phylogenetic signal (*λ*) of the variables and estimated simultaneously on the regression models’ residuals, as Revell (2010) suggested

Variable	*λ*	*P*-value	Regression model	*λ*	*P*-value
% ZW	1.00	0.0001	Total GS ∼ d_N_/d_S_	1.01	0.062
d_N_/d_S_	0.00	1.00	d_N_/d_S_ ∼ % ZW	−0.30	0.332
Total GS	1.00	0.173	Total GS ∼ % ZW	1.00	0.031
Intergenic GS	0.00	1.00	Intergenic GS ∼ % ZW	0.64	0.276
*u*	1.00	0.057	Total GS ∼ *u*	1.00	0.039
S0 genes lost	0.97	0.001	S0 genes lost ∼ % ZW	0.94	0.001

*P*-values are from hypothesis tests for a significant phylogenetic signal against the null model (*λ* = 0).

In addition to an elevated accumulation of nonsynonymous mutations, a reduction in the efficacy of selection is also predicted to correspond with increased occurrences of radical amino acid changes, particularly those altering physicochemical properties like charge (Miyata et al. 1979), as these are more likely to affect protein function and be deleterious than conservative, within-group, changes (Hanada et al. 2007). Indeed, binomial logistic regression revealed a positive correlation between % ZW differentiation and the occurrence of radical amino acid changes (Fig. 2b). Specifically, we estimate that the log odds of an observed amino acid change being radical increased by 0.47% with each 1% increase in ZW differentiation (*P* = 0.044).

Additionally, we investigated whether ZW differentiation affects the *N*_e_ of the W chromosome itself. To address this, we examined the retention and loss patterns of genes within the ancestrally differentiated region of the chromosome (the oldest, “S0” stratum, as identified by Xu and Zhou 2020). The S0 stratum, shared by all birds, represents the most evolutionarily ancient region of the W chromosome and is characterized by high sequence divergence and gene loss, reflecting its long history of independent evolution. Expanded ZW differentiation predicts increased HRI effects and thus decreased *N*_e_ for the S0 region. Consistent with increased HRI effects, species with higher % ZW differentiation experienced a more significant loss of S0 genes (using OLS regression: *β* = 36.6, *P* = 0.0004; Fig. 2c). However, in this case, phylogenetic nonindependence due to shared ancestry creates a statistical issue that should be accounted for (Table 1), so we also used PGLS regression and found the relationship remains significant (*β* = 24.0, *P* = 0.044).

### Lack of Evidence for a Relationship Between Effective Population Size and Mitochondrial GS

**Fig. 3.**
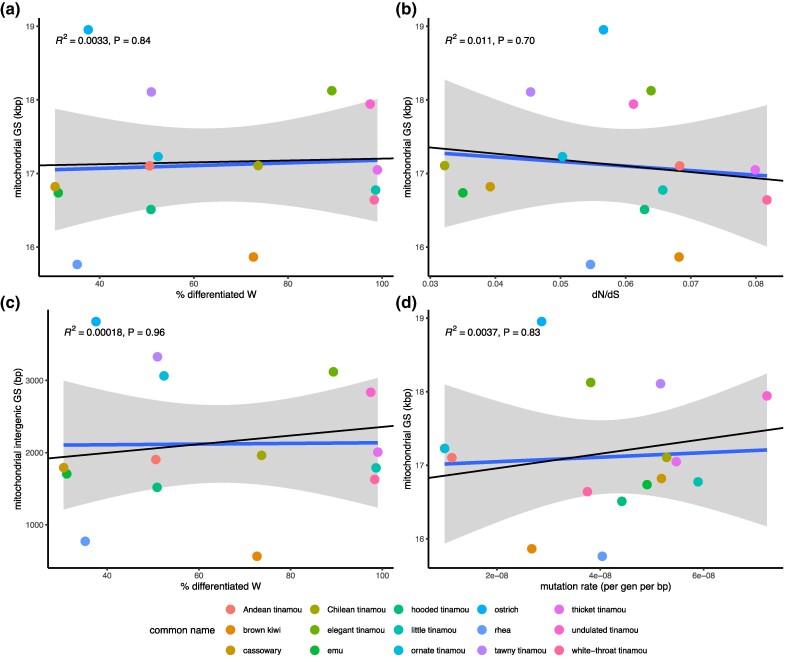
Correlations between the d_N_/d_S_ ratio, mitochondrial GS, and mutation rate. Blue lines represent the regression coefficient (*β*) from OLS regression, and black lines are from PGLS regression in 15 species of palaeognathous birds. a) No reliable correlation exists between d_N_/d_S_ and mitochondrial GS. b) There is no reliable correlation between the amount of genetic linkage and mitochondrial GS. c) There is no significant relationship between the amount of intergenic DNA and gene linkage. d) No significant relationship is observed between mitochondrial GS and the per-generation mutation rate per nucleotide site (*u*).

If *N*_e_ significantly influences GS, the observed relationship between ZW differentiation and *N*_e_ of the mitochondrion predicts a positive relationship between ZW differentiation and mitochondrial GS. However, we found no correlation between % W chromosome differentiation and mitochondrial GS, with neither PGLS (*β* = 0.12, *P* = 0.93) nor OLS regression (*β* = 0.186, *P* = 0.84) demonstrating any significant effect (Fig. 3a), nor between mitochondrial GS and d_N_/d_S_ (*β* = −6.16, *P* = 0.70; Fig. 3b). We also examined the relationship between the amount of intergenic mitochondrial DNA and % W chromosome differentiation. We again found no significant relationship with either PGLS (*β* = 5.99, *P* = 0.83) or OLS regression (*β* = 0.471, *P* = 0.96; Fig. 3c). Our analysis suggests that changes in *N*_e_ do not correlate with changes in mitochondrial GS in palaeognathous birds, contrary to the predictions of the MHH.

### Lack of Evidence for a Relationship Between Mitochondrial Mutation Rate and Mitochondrial GS

Our data set also allows us to test an additional proposed determinant of GS: *u*. According to Lynch et al.’s (2006) model of organelle GS evolution, populations with higher *u* experience stronger selection against maladaptive increases in GS than those with lower *u*. To obtain estimates of per-generation mitochondrial *u*, we first calculated the number of generations represented by each terminal branch length as the estimated branch length in years divided by the extant species’ estimated generation time. The reliability of fossil records in early-branching birds has been well scrutinized (Jarvis et al. 2014; Prum et al. 2015; Yonezawa et al. 2017), and divergence time estimates are highly reliable and consistent, regardless of the taxon sampling and fossil calibrations used. We then computed per-generation mutation rates by dividing the estimated d_S_ value by the estimated number of generations. It is important to note that while the nuclear mutation rate influences branch lengths, the mitochondrial mutation rate does not affect them. As these two parameters are governed by entirely different molecular machinery, we do not expect a high degree of circularity when estimating mitochondrial *u*. We found no significant relationship using PGLS (*β* = 9.9e06, *P* = 0.49) or OLS (*β* = 3.1e06, *P* = 0.83) (Fig. 3c). Contrary to the predicted direction, the association was nonsignificantly positive, with species with higher mutation rates tending to have larger genomes. These findings challenge the predictions of the MHH and highlight the complex interplay of factors likely shaping GS in palaeognathous birds.

## Discussion

Understanding the forces governing GS has long captivated researchers. At the forefront of this debate is the idea that nonessential genomic insertions are slightly deleterious, at least under some circumstances, allowing them to fix and persist in some genomes while being excluded from others. However, empirical tests of this hypothesis have been challenging due to the multitude of potential factors influencing selection intensity against these elements (Charlesworth and Barton 2004). Additionally, the technical complexities of quantitatively estimating these factors pose significant hurdles (Waples et al. 2013). Our study provides a rare, relatively direct test of the slightly deleterious genome expansion hypothesis, relying on a theoretically and empirically supported decrease in selective efficiency resulting from increased genetic linkage.

Our first major finding confirms the predicted association between increased ZW sex chromosome differentiation and reduced *N*_e_ in genetically linked mitochondrial and sex chromosomal genomic regions. While the predicted consequence of increased ZW differentiation is a decrease in *N*_e_ due to greater HRI under increased genetic linkage, confidently inferring this causality remains challenging as alternative explanations are plausible. Notably, a decrease in population *N*_e_ could drive ZW differentiation and mitochondrial *N*_e_. Some may interpret our results in terms of HRI without invoking *N*_e_. However, HRI and *N*_e_ are interconnected: *N*_e_ is defined as nucleotide diversity (π) over *u*, and HRI reduces π, thereby implying changes in *N*_e_. Since HRI is one factor that influences *N*_e_, differences in *N*_e_ due to varying levels of HRI are pertinent to testing hypotheses about its overall effects. Our test relies on predicted directional differences in the magnitude of *N*_e_ and the efficacy of selection rather than on specific estimates of *N*_e_.

Our second significant finding is that the GS of the mitochondrion is not associated with the *N*_e_ or *u* of the mitochondrion. To clarify the relevance of both *u* and *N*_e_ in testing hypotheses related to genome evolution, it is worth noting that the parameter *N*_e_ × *u* is consistently emphasized in population genetics, as it represents the combined influence of genetic drift and mutation rate, which is central to understanding the balance between the introduction of new mutations and the efficiency of selection in removing deleterious alleles. Fundamentally, the MHH claims that “mutationally hazardous” DNA is more likely to accumulate in species with a small *N*_e_ and low *u* when genetic drift is high than those with high *N*_e_ and *u* when drift is minimal. While previous work often focuses on only one of the parameters—highlighting *N*_e_ when discussing nuclear genomes and *u* when discussing mitochondrial genomes—the emphasis on one variable over the other is a matter of choice by previous authors rather than a fundamental issue with the underlying theory. Our failure to find a relationship between GS and *N*_e_ or *u* is inconsistent with the prediction of the MHH hypothesis and the broader hypothesis that genomic expansion incurs a fitness cost. Given the long-standing challenges in enacting controlled tests of this hypothesis and the relatively straightforward nature of the natural experiment used here, our results suggest a need for a more sustained effort to assess the predictive power of the MHH and the idea that increased GS and complexity are, by and large, slightly deleterious.

However, two potentially important objections may be raised to our approach. First, mitochondrial GS shows little variation across the studied taxa and thus may not represent an ideal data set. While it is true that total variation in GS is moderate, there is substantial variation in the overall contents and structure of the genome, from the highly streamlined genome of *Apteryx mantelli*, where the core coding sequences account for the vast majority of the genome (96%), to *Struthio camelus*, in which intergenic DNA makes up nearly a quarter of the genome (20%). However, the fact that these taxa have relatively slight variation in mitochondrial GS despite varying *N*_e_ itself rejects the MHH, given that it predicts variation under these circumstances. It may be that bird mitochondrial GS is governed by a cryptic lineage-specific tendency toward smaller GS, driven by the energetic demands of flight (Wright et al. 2014), or, in the case of the semiflighted or flightless birds studied here, the high metabolic cost of running (Bundle et al. 1999). However, again, these contentions oppose the central claim of the MHH, namely that GS variation is primarily explained by *N*_e_ or *u* (Lynch and Conery 2003). Asserting that a hypothesis possesses explanatory power only under specific post hoc circumstances implies that its overall explanatory capacity remains limited at best.

A second question concerns the extent of the change in *N*_e_ arising from the expanded W chromosomal region. Estimating such values requires extensive knowledge of the distribution of selective effects of newly arising nonsynonymous mutations in the mitochondrial genome. While the increases in d_N_/d_S_ may be seen as somewhat moderate (around 1.5-fold between short-W and long-W species), widespread degradation of W chromosome regions in birds and other lineages (involving gene loss, increased d_N_/d_S_, and the massive accumulation of transposable elements) suggests substantial reductions in the efficiency of selection (Wang et al. 2021; Warmuth et al. 2022).

Our investigation represents a rare opportunity to explore a relatively well-controlled instance of the intricate relationship between genetic drift, mutation rate (*u*), and organelle GS. By focusing on a controlled case where variation in *N*_e_ arises from genetic changes in a single genomic region (rather than global demographic shifts), we confirm the expected changes in *N*_e_ through standard measures of selective efficiency. To transcend the specific framing of any single hypothesis, our study tested a broader prediction: if mitochondrial GS expansions are slightly deleterious, their fixation should increase as *N*_e_ or *u* decrease. However, our failure to find the expected associations underscores the limitations of the MHH and other explanations positing weak or inefficient selection on expanded genomes, highlighting the need for further controlled tests.

## Materials and Methods

We estimated the mitogenome-wide d_N_/d_S_ ratio using the FitMG94 workflow in HyPhy v. 2.5.36 (https://github.com/veg/hyphy-analyses/tree/master/FitMG94; Pond and Muse 2005), including credible intervals. The MG94 codon evolution model incorporates synonymous and nonsynonymous nucleotide substitution rates as parameters, correcting for multiple hits at a codon and allowing d_S_ to vary across branches (Muse and Gaut 1994). Additionally, the FitMG94 workflow employs a corrected empirical estimator (CF3 × 4), which provides improved estimates of several parameters from a standard model. This estimator accounts for individual nucleotide frequencies at three codon positions and corrects for biases induced by stop codons (Goldman and Yang 1994).

To validate our HyPhy estimates, we compared them to d_N_/d_S_ estimates obtained using the free-ratio model in PAML (Yang 2007) and found they largely agreed. However, PAML lacks a formal method for calculating credible intervals. Given the small sample size and considerable uncertainty associated with both methods for the two short-branch sister taxa, we opted to proceed with the HyPhy estimates. For the same reasons, we implemented Bayesian linear and mixed phylogenetic models incorporating the uncertainty estimates for d_N_/d_S_. We present the latter here since our Bayesian approaches yielded qualitatively similar results to likelihood methods. Results and information on the Bayesian analyses are available in the Supplementary material. Generation times used for estimating *u* were sourced from Wang et al. (2021) unless otherwise specified.

To estimate ancestral states for % ZW differentiation, we used the fastAnc (fast estimation of ML ancestral states) function in phytools (Revell 2012). We used aaML in PAML without rate variation to infer ancestral mitogenome sequences. We then calculated radical and conservative amino acid changes across all internal and external branches using RadAA (Seim et al. 2019), which identifies pairwise amino acid changes in multiple sequence alignments and categorizes residues into groups based on their charge, with cysteine forming its own group. The lengths of tRNA genes were calculated using Arwen (Laslett and Canbäck 2008) and tRNAscan-SE (version 2.0; Chan et al. 2021), while the ribosomal RNA genes (rrnL and rrnS) were annotated using DeGeCI (version 1.1; Fiedler et al. 2024).

We computed individual variables’ phylogenetic signal (*λ*) using the phylosig function in phytools (Revell 2012). To validate the use of phylogenetic correction for linear regressions, we followed Revell (2010) instructions to simultaneously estimate Pagel's lambda (*λ*) with the linear regression using the “corPagel” and gls function in nlme (Pinheiro et al. 2018). *P*-values for the significance of the phylogenetic signal in the residuals were obtained using an ANOVA test comparing our *λ* model with a model that has *λ* fixed at zero. Unless specified in the Supplementary material, all statistical analyses were performed using R (v4.3.1).

## Data Availability

The primary data underlying this article are available on Github at https://github.com/Brookesloci/Paleognath_ZW_GS.

## Supplementary Material

Supplementary material is available at https://github.com/Brookesloci/Paleognath_ZW_GS.
